# A bacteriocin gene cluster able to enhance plasmid maintenance in *Lactococcus lactis*

**DOI:** 10.1186/1475-2859-13-77

**Published:** 2014-05-28

**Authors:** Ana B Campelo, Clara Roces, M Luz Mohedano, Paloma López, Ana Rodríguez, Beatriz Martínez

**Affiliations:** 1 Dairy Safe group, Department Technology and Biotechnology of Dairy Products, Instituto de Productos Lácteos de AsturiasIPLA-CSIC, Paseo Río Linares, s/n, 33300 Villaviciosa, Asturias, Spain; 2 Departamento de Microbiología Molecular y Biología de las Infecciones, Centro de Investigaciones Biológicas CIB (CSIC), 28040 Madrid, Spain

**Keywords:** Plasmid segregation, Post-segregational killing, Bacteriocins, Insertion sequences, Lactococcus lactis

## Abstract

**Background:**

*Lactococcus lactis* is widely used as a dairy starter and has been extensively studied. Based on the acquired knowledge on its physiology and metabolism, new applications have been envisaged and there is an increasing interest of using *L. lactis* as a cell factory. Plasmids constitute the main toolbox for *L. lactis* genetic engineering and most rely on antibiotic resistant markers for plasmid selection and maintenance. In this work, we have assessed the ability of the bacteriocin Lactococcin 972 (Lcn972) gene cluster to behave as a food-grade post-segregational killing system to stabilize recombinant plasmids in *L. lactis* in the absence of antibiotics. Lcn972 is a non-lantibiotic bacteriocin encoded by the 11-kbp plasmid pBL1 with a potent antimicrobial activity against *Lactococcus*.

**Results:**

Attempts to clone the full *lcn972* operon with its own promoter (P_972_)_,_ the structural gene *lcn972* and the immunity genes *orf2-orf3* in the unstable plasmid pIL252 failed and only plasmids with a mutated promoter were recovered. Alternatively, cloning under other constitutive promoters was approached and achieved, but bacteriocin production levels were lower than those provided by pBL1. Segregational stability studies revealed that the recombinant plasmids that yielded high bacteriocin titers were maintained for at least 200 generations without antibiotic selection. In the case of expression vectors such as pTRL1, the Lcn972 gene cluster also contributed to plasmid maintenance without compromising the production of the fluorescent mCherry protein. Furthermore, unstable Lcn972 recombinant plasmids became integrated into the chromosome through the activity of insertion sequences, supporting the notion that Lcn972 does apply a strong selective pressure against susceptible cells. Despite of it, the Lcn972 gene cluster was not enough to avoid the use of antibiotics to select plasmid-bearing cells right after transformation.

**Conclusions:**

Inserting the Lcn972 cluster into segregational unstable plasmids prevents their lost by segregation and probable could be applied as an alternative to the use of antibiotics to support safer and more sustainable biotechnological applications of genetically engineered *L. lactis.*

## Background

*Lactococcus lactis* is widely used as starter in the manufacture of cheese. It belongs to the diverse group of lactic acid bacteria which are characterized by the production of large quantities of lactic acid as a consequence of the fermentative metabolism of carbohydrates. Due to its main role in food fermentation, *L. lactis* has been extensively studied and has become a model lactic acid bacterium. The deep knowledge of *L. lactis* physiology along with the advent of high-throughput technologies (−omics) has paved the way for novel biotechnological applications of this species [1]. Among them, it is worth mentioning several metabolic engineering strategies to re-route *L. lactis* metabolism to the production of metabolites of interest for both food and pharmaceutical industries. *L. lactis* has been engineered to produce commodity chemicals such as mannitol and 2,3-butanediol *ex situ,* i.e. as a cell factory or to produce vitamins *in situ*[2,3]. *L. lactis* is also a suitable platform for the production of recombinant proteins [4,5].

To address these challenges, several genetic tools have been devised that expand from replicative cloning plasmids to chromosomal integration strategies, (inducible-) gene expression platforms and synthetic promoters, among others [1,5]. Plasmid-based approaches have already been exploited since 25 years ago and still comprise the main core of the *L. lactis* toolbox [6]. Most of the lactococcal vectors rely on antibiotic resistance markers, namely erythromycin and chloramphenicol, which are needed for cloning and to stably maintain the plasmid during bacterial growth. However, in the context of biosafety, the use of antibiotics hinders large scale fermentations by increasing costs. Also, the risk of end-product contamination with the antibiotic is higher, and containment measurements to avoid spread of antibiotic resistance genes are required [7,8]. Consequently, alternatives to antibiotic selection have been approached based on the complementation of auxotrophies and mutations in metabolic genes [9-12]. The use of antimicrobial peptides produced by bacteria (i.e. bacteriocins) as selecting agents and their cognate immunity genes as selectable marker genes have also been explored as food-grade cloning strategies. The nisin immunity gene *nisI* has been incorporated into plasmids that could be selected with nisin in *Lactococci* and *Lactobacilli*[13]. Moreover, lacticin 481 and lacticin 3147 conjugative plasmids could be easily transferred in mating experiments by selection of transconjugants with the corresponding bacteriocin [14,15]. These examples support the potential of bacteriocin-based approaches to develop food-grade genetic tools.

In this work, we have assessed the use of the Lcn972 gene cluster to improve the segregational stability of lactococcal plasmids without the aid of antibiotics. Lcn972 is a non-lantibiotic bacteriocin that selectively inhibits cell wall biosynthesis in *Lactococci* by binding to the cell wall precursor lipid II [16,17]. Lcn972 is encoded by the theta-replicating 11 kbp-plasmid pBL1 [GenBank:AF242367.1]. The Lcn972 biosynthetic machinery comprises the structural gene *lcn972* and two genes, *orf2* and *orf3* which could encode a putative ABC transporter involved in immunity [18,19]. Shuttle *E. coli-Lactococcus* plasmids based on the pBL1 replicon revealed a high intrinsic instability, unusual for theta-replicating plasmids, and suggested that production of Lcn972 could be involved in postsegregational killing and, thereby, contribute to plasmid maintenance [18,19]. On these premises, the segregational stability in the absence of antibiotics of different recombinant plasmids bearing the Lcn972 gene cluster has been evaluated.

## Results and discussion

### Cloning of the *lcn972* cluster in the pIL252 replicon

**Figure 1:**
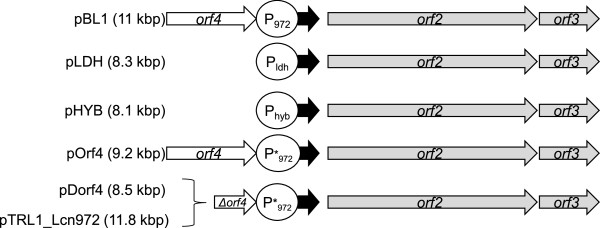
**Schematic drawing of the plasmids bearing the Lcn972 gene cluster.** The plasmids are based on the pIL252 replicon (4.6 kbp) besides pTLR1_Lcn972, which is based on pTLR1, and pBL1, which is the original plasmid found in the wild-type Lcn972-producing strain *L. lactis* IPLA972. Black arrow: structural *lcn972* gene; grey arrows: immunity genes; white arrow: hypothetical *orf4*. The different promoters driving expression of the Lcn972 gene cluster are shown in circles. P*_972_: mutated promoter. The size of each plasmid is shown in brackets.

Previous reports suggested a putative role of the Lcn972 gene cluster in the maintenance of the wild-type Lcn972-plasmid pBL1. First, attempts to cure pBL1 in the wild-type Lcn972 producer *L. lactis* IPLA972 failed [18]. Secondly, the pBL1 replicon alone was segregationally unstable and was quickly lost in the absence of antibiotic selection. Moreover, pBL1 transfer to *L. lactis* by conjugative mobilization also failed due to the potent antimicrobial activity of donor cells on *L. lactis* recipient cells [19]. Overall, these results supported the notion that the Lcn972 gene cluster might be involved in plasmid maintenance by post-segregational killing, i.e. eliminating those cells which have lost the plasmid during division due to the strong toxicity of the bacteriocin. To demonstrate if this was the case, we selected the plasmid pIL252, which has been shown to easily segregate in *L. lactis*[20,21], to clone the Lcn972 gene cluster consisting of the structural gene *lcn972* and the putative genes involved in immunity (*orf2-orf3*) (Figure 1).

Several attempts to clone the Lcn972 gene cluster with its own P_972_ promoter repeatedly failed and transformants were never recovered. Alternatively, the promoterless Lcn972 gene cluster was cloned in the vector pIL252 under the control of two available promoters: P_ldh_ and P_hyb_ (Figure 1). These promoters were chosen on the basis of their reported constitutive expression and high activity [22,23]. The *in vitro* constructed recombinant pLDH and pHYB plasmids were easily introduced into *L. lactis* NZ9000 and preliminary testing confirmed the production of Lcn972.

Once the Lcn972 gene cluster was cloned in pIL252, attempts to introduce the original promoter P_972_ were approached and two other plasmids pOrf4 and pDorf4 were constructed (Figure 1). pOrf4 included *orf4*, an ORF of unknown function [GenBank:NP_862431], upstream of the Lcn972 gene cluster in pBL1. It is common that self-protection in bacteriocin producers rely on more than one immunity system as it is the case of several lantibiotics such as nisin and lacticin 3147 [24,25] and the circular bacteriocin AS-48 [26]. Therefore, we speculated that *orf4* might be required to enhance survival of Lcn972 producing cells and, consequently, facilitate cloning of the whole Lcn972 gene cluster. However, when we tried to generate pOrf4, only one transformant was recovered. Sequencing of pOrf4 revealed the presence of a single nucleotide insertion (A, at position 3126 referred to pBL1 [GenBank:AF242367.1] coordinates) between the −35 (TTGACA) and −10 (TAAAAT) sequences of P_972_. Such mutation might interfere with transcription since the length of the spacer sequence between the −35 and the −10 sequences often determines promoter strength in *L. lactis*[27,28]. However, *L. lactis* pOrf4 was still able to inhibit the growth of the indicator strain in the direct antagonism test (data not shown), indicating that Lcn972 was synthesized despite of the mutation. From pOrf4, another plasmid pDorf4 was generated by deleting the 5’ end of *orf4* (see Figure 1) to check if there was any effect on bacteriocin titers or immunity in the absence of *orf4* as described below.

### Growth and bacteriocin production

Growth and bacteriocin production by all the pIL252 recombinant plasmids in *L. lactis* NZ9000 were assessed (Table 1). To compare their behaviour in terms of growth and bacteriocin production, *L. lactis* NZ9000 carrying pBL1 was used as a reference.

**Table 1:** **Growth and bacteriocin production of *L. lactis *** **NZ9000 transformed with Lcn972-expressing plasmids**

**Plasmid**	**μ (h**^**−1**^**)**	**g (h)**	**ODmax**	**Lcn972 activity (AU**^**a**^**/mL)**	**MIC**^**b **^**Lcn972 (AU/mL)**
pBL1 (WT)	1.04 ± 0.13	0.67 ± 0.08	3.52 ± 0.21	6400	1600
pLDH	1.02 ± 0.12	0.69 ± 0.08	3.44 ± 0.37	800	1600
pHYB	0.67 ± 0.06	1.04 ± 0.09	3.18 ± 0.47	200	1600
pOrf4	1.01 ± 0.10	0.69 ± 0.07	3.25 ± 0.36	400	1600
pDorf4	0.88 ± 0.04	0.79 ± 0.03	3.45 ± 0.30	400	800

^a^AU: Arbitrary Units.

^b^MIC: Minimal Inhibitory Concentration. The MIC of Lcn972 for *L. lactis* NZ9000 is 12.5 AU/mL.

Growth rate (μ), generation time (g), maximal OD_600_ (ODmax) and production and immunity to Lcn972 are shown. Results are the average ± standard deviation from, at least, two independent growth experiments in GM17.

Growth kinetics of *L. lactis* carrying pLDH, pOrf4 or pBL1 showed similar growth rates, generation times and maximal OD (Table 1). *L. lactis* pDorf4 grew slightly slower but reached similar maximal OD as the others. On the contrary, cultures carrying the fusion of the Lcn972 cluster to the promoter P_hyb_ had impaired growth and the growth rate was only 64% of that shown by the reference *L. lactis* with pBL1. Also, the maximal ODs reached at the end of the exponential phase were lower in *L. latis* pHYB cultures (Table 1).

Bacteriocin production was detected in the supernatant of all the cultures (Table 1) and was maximal at the end of the exponential phase, as previously described for pBL1 cultures [18] albeit bacteriocin titers encoded by pIL252 derivatives were lower than those provided by pBL1. On the other hand, Lcn972 activity provided by pHYB was 4 times lower than that by pLDH, likely reflecting the strength of each promoter (Table 1). Bacteriocin production by pOrf4 and pDorf4 plasmids was also lower than pBL1. Both recombinant plasmids reached similar titers regardless of the existence of *orf4.* Therefore, this gene does not seem to participate in Lcn972 biosynthesis or to provide additional immunity and the reason why the Lcn972 gene cluster with its own promoter could not be cloned in pIL252 remains unanswered.

### Segregational stability of Lcn972-recombinant plasmids

**Figure 2:**
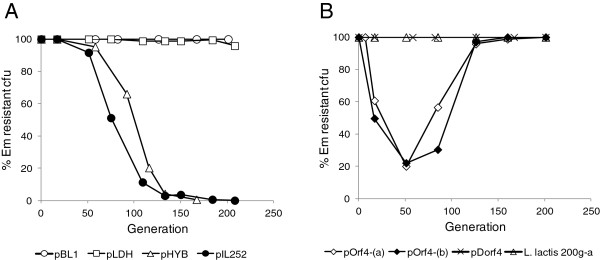
**Segregational stability of Lcn972-expressing plasmids in *L. lactis *NZ9000 grown without erythromycin (Em).** All the experiments were started from fresh overnight cultures in GM17Em (except pBL1). Experiments were carried out once except for pIL252 (in **A**), that was performed three times (only one representative curve is shown), and for pOrf4 (in **B**), where two independent Em-resistant colonies (−a, −b) were tested. *L. lactis* 200 g-a is a Em-resistant Lcn972-producing colony isolated from *L. lactis* pOrf4 after 200 generations.

In order to check if the Lcn972 gene cluster could enhance maintenance of the plasmids without antibiotic selection, *L. lactis* cultures carrying the recombinant plasmids were sub-cultured for 200 generations in GM17 and the fraction of erythromycin (Em)-resistant and Lcn972 producing colonies was calculated at time intervals (Figure 2). *L. lactis* pBL1 and pIL252 were also examined as references.

In agreement with previous reports, the plasmid pIL252 was quickly lost [20,21], as judged by the loss of Em-resistance, whilst pBL1 remained stable and all the tested colony forming units (cfu) were able to produce Lcn972, confirmed by the presence of inhibition halos on *L. lactis* MG1614 plates (data not shown). The plasmid pLDH was also segregationally stable although 4.2% of the cells had lost the plasmid after 200 generations. The presence of the Lcn972 gene cluster under the control of the P_hyb_ promoter slightly delayed segregation but it was finally lost after 167 generations (Figure 2A).The behavior of pOrf4 was radically different. Plasmid loss occurred quickly and after 50 generations only 20% of the isolated colonies were Em-resistant and Lcn972-producers (Figure 2B). Surprisingly, the percentage of Em-resistant colonies increased thereafter, and a supposedly homogenous Em-resistant population was recovered after 150 generations in which, all the tested colonies were positive in the bacteriocin activity test. This particular behavior was reproducible and repeating the experiment with another independent clone gave similar results (Figure 2B).

The deletion of the 705 bp downstream of the 5’ cloning site of pOrf4 to make pDorf4 largely enhanced plasmid stability to such extent that plasmid segregation was not longer observed (Figure 2B). To discard any fortuitous mutations and confirm that the 3.5 kbp DNA insert of pDorf4 could indeed stabilize pIL252, this DNA fragment was amplified by PCR, using as template pDorf4 and primers SPCf and SPCr (Table 2) and cloned in pIL252. Transformants were easily obtained and the newly constructed pDorf4 plasmid was as stable as pDorf4 created after deleting the *Eco*RI-*Eco*RV 705 bp-fragment from pOrf4.

**Table 2:** Primers used in this work

**Primer**	**Sequence 5’-3’ (restriction site)**	**Position**
orf4-F	CCGCTG**GAATTC**TCAAACG (*Eco*RI)	5’ *orf4* in pBL1
orf4-R	CACCGG**CACGTTGTG**TATCC (*Dra*III)	3’ *lcn972*
ery5	GAAGATCTAGATATAATGGGAGATAAGACGG	5’ *ery* in pIL252
SPCf	GCGGATCCGATATC**GAATTC**TGTAGACCCTCCG (*Eco*RI)	5’ Δ*orf4* in pDorf4
SPCr	CG**GAATTC**CCGGGCTCGAGTCTAGAG (*Eco*RI)	3’ *orf3* in pDorf4
lcn2	CTTGTTAAAAGAATTCGCTGCAAACC	3’ *orf2* in pBL1
eryrepF-F	GCGCTTAGAATCGCTTTAGG	intergenic *ery-repF*
repE-F	GATTGGCGGTCGTGGTGTTG	5’ *repE*
repE-R	GTCACATAGACTGGCGTTTC	*repE*
repE2-R	TAGGGCGTTCTGCTAGCTTG	3’ *repE*
201 g-F	GAGCTAAAGAGGTCCCTAGCGCTTAGAATC	5’ *repF*
201 g-R	GAAGTCAGAACAACACCACGACCGCCAATC	intergenic *ery-repF*
402 g-F	GGCGGTCGTGGTGTTGTTCTGACTTCC	*repE*
402 g-R	TTCCTGATTCGCCTTCAGTACCTTCAGC	*repE*

These results support post-segregational killing as the most likely mechanism behind plasmid stabilization by the Lcn972 gene cluster because those recombinant plasmids producing high Lcn972 levels such as pLDH and pDorf4 (800–400 AU/mL) were kept in the population, while pHYB (200 AU/mL) was quickly lost. Post-segregational killing by bacteriocin-encoding plasmids in Gram positives has been demonstrated in pLgLA39, a plasmid encoding the circular bacteriocin gassericin A in *Lactobacillus gasseri* LA39. Deletion of the gassericin A structural gene in gassericin A-expressing plasmids significantly reduced plasmid stability [29].

### The Lcn972 gene cluster stabilizes mCherry-expressing vectors

**Figure 3:**
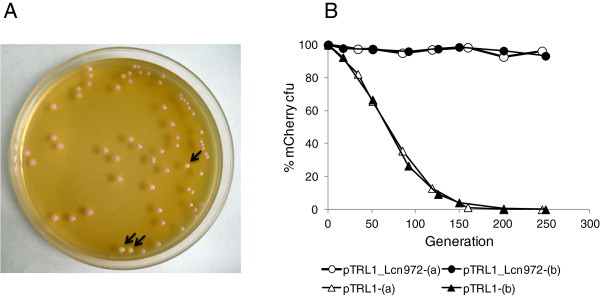
**Segregational stability of the red fluorescent protein mCherry expression plasmid pTRL1 with and without the Lcn972 gene cluster. (A)** Pink colony phenotype of pTRL1 to assess the functional expression of the mCherry protein in *L. lactis* NZ9000 cells. The arrows point to plasmid-free colonies. **(B)** Stability of the plasmid pTRL1 and pTRL1_Lcn972 in which the Lcn972 gene cluster from pDorf4 was cloned. Cultures were grown in GM17 without erythromycin and the presence of the plasmids was determined by pink/white screening on an average of 390 colonies per sampling time. The experiments were carried out with two independent cultures denoted as (a) and (b).

Further confirmation of the contribution of the Lcn972 gene cluster to plasmid maintenance was obtained by evaluating the loss of the expression vector pTLR1 in the absence of antibiotic selection. pTLR1 is a shuttle vector in which the *mrfp* gene, encoding the red fluorescent protein mCherry, is under the control of the constitutive Px promoter and it also carries the erythromycin resistance gene *erm*[30]. This plasmid was chosen because the synthesis of mCherry could be easily assessed as plasmid-bearing colonies develop a pale pink colour on GM17 plates due to the functional expression of mCherry (Figure 3A). Moreover, fluorescent proteins are becoming the reporter of choice for *in vivo* gene expression studies, for example, during bacterial infection or gut colonization, where antibiotics cannot be present [31].The 3.5 kbp Lcn972 gene cluster was cloned in pTRL1 to create pTRL1_Lcn972. Production of Lcn972 was confirmed by the direct antagonism test (data not shown) and the stability of both plasmids was assessed (Figure 3B). Similar to the reference plasmid pIL252, pTRL1 was lost after 150 generations. On the contrary, pTRL1_Lcn972 was stable and maintained after 250 generations, validating the use of Lcn972 gene cluster to stabilize expression plasmids without compromising the synthesis of reporter proteins.

### Insertion sequences are involved in the integration of pOrf4 into the chromosome

To explain the particular behavior of the plasmid pOrf4 in *L. lactis* NZ9000, three independent colonies (*L. lactis* 200 g-a, −b and -c) were selected once the population was stable. *L. lactis* 200 g-a was further sub-cultivated for another 200 generations and remained resistant to Em and produced Lcn972. One colony, *L. lactis* 400 g, i.e. *L. lactis* pOrf4 after 400 generations, was randomly selected and used in subsequent experiments.

**Figure 4:**
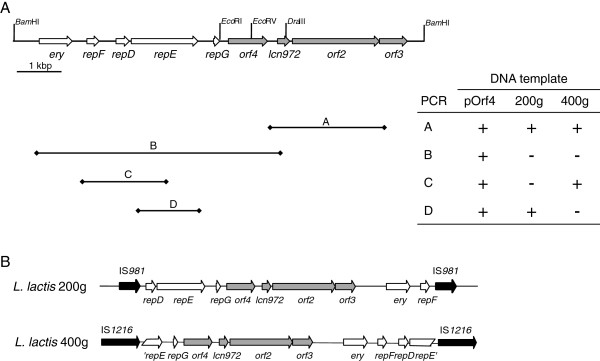
**Integration of the plasmid pOrf4 in the chromosome of *L. lactis *NZ9000. (A)** Results of the PCR strategy to define the region of pOrf4 involved in the molecular reorganization with the chromosome. Primer pairs orf4-F/lcn2, ery5/orf4-R, eryrepF-F/repE-R, and repE-F/repE2-R were used in PCRs A, B, C and D, respectively. **(B)** Presence of the insertion sequences (black arrows) IS*981* and IS*1216* as revealed by direct sequencing of the chromosome of *L. lactis* 200 g and *L. lactis* 400 g, respectively.

Plasmid DNA could not be isolated from any of the *L. lactis* 200 g or *L. lactis* 400 g cultures grown on GM17Em, suggesting that the plasmid might have been integrated into the chromosome. A set of PCRs with primers annealing on the plasmid was designed to determine the fragments of pOrf4 that were involved in rearrangements with the chromosome (Figure 4A). It was determined that recombination had taken place within the intergenic region between *repF* and *repD* in *L. lactis* 200 g, and within the coding region of *repE* in *L. lactis* 400 g (Figure 4A). Moreover, direct sequencing on the chromosome with primers annealing on the plasmid and pointing towards both ends of the integration site revealed the presence of the insertion sequence IS*981* at either side of the integrated plasmid pOrf4 in *L. lactis* 200 g and of IS*1216* in *L. lactis* 400 g (Figure 4B).

The intrinsic instability of the plasmid pOrf4 must be due to the presence of the DNA sequence from the stop codon of *repG* to the internal *Eco*RV site in *orf4*. This region is absent in pDorf4 which is stably maintained as a plasmid. This particular DNA region is also present in the *L. lactis* plasmid pLP712 (98.7% identity in BlastN searches at NCBI) and it has been described as a hotspot for DNA rearrangements through the activity of insertion sequence elements as several deletion derivatives of pLP712 occur at this site [32]. The presence of insertion sequences flanking the integrated pOrf4 plasmid also supports the contribution of these mobile elements that led to the integration of the pOrf4 plasmid.

Bacteriocin production by the pOrf4 integrants *L. lactis* 200 g and *L. lactis* 400 g was 200 AU/mL (data not shown). The bacteriocin titers are lower than those provided by the plasmid pOrf4 (Table 1), and may be explained by a reduced gene dosage as a consequence of integration. Still, the levels of Lcn972 were high enough to displace susceptible cells, further supporting the strong selection pressure exerted by this bacteriocin.

### Selection of transformed lactococcal cells with Lcn972-recombinant plasmids after electroporation

**Figure 5:**
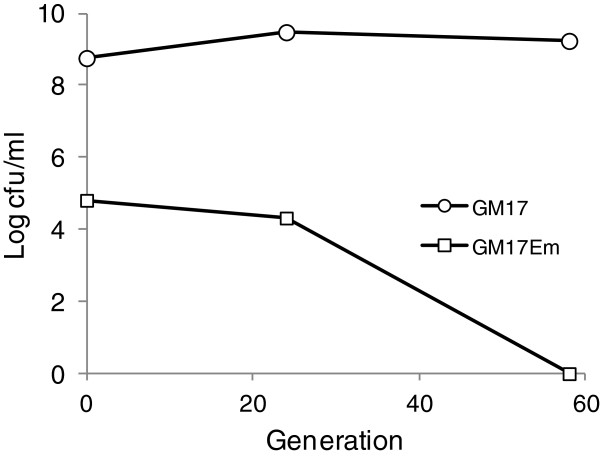
**Presence of the Lcn972 gene cluster does not allow direct selection of transformants after plasmid electroporation.***L. lactis* NZ9000 was electroporated with pDorf4 and colony forming units of the transformation mix right after electroporation (generation 0) and during sub-culturing in GM17 were followed by plating in the presence (GM17Em) or absence (GM17) of erythromycin.

Genes involved in resistance and immunity to bacteriocins from Gram positive bacteria have been claimed to facilitate selection of recombinant plasmids using the cognate bacteriocin as a selecting agent instead of antibiotics [13-15,33]. Lcn972 was also useful to select pBL1-bearing *L. lactis* cells after electroporation with the plasmid content of the wild-type producer *L. lactis* IPLA972 [18]. However, Lcn972 is a thermosensitive bacteriocin which makes its use as a selecting agent not very accurate as it might be inactivated during the preparation of selecting plates. Nevertheless, in view of the strong selective pressure that Lcn972 seems to apply on *L. lactis*, we checked if lactococcal cells that acquired plasmids bearing the Lcn972 cluster could compete against plasmid-free cells right after electroporation so that antibiotics, and consequently antibiotic resistance markers, would not be further required to select positive transformants. To test it, *L. lactis* NZ9000 was transformed with pDorf4 and after 90 min of incubation ten-fold dilutions were plated on GM17 with and without Em. After transformation, 0.01% of the cells was resistant to erythromycin, and regarded as true transformants carrying the plasmid (Figure 5). The transformation mix was subsequently sub-cultured in GM17 broth and transferred every 7–8 generations to fresh broth. Appropriate dilutions were also plated on GM17 and GM17Em to estimate the fraction of the population that still retained the plasmid. As shown in Figure 5, while there were still 4.3 log cfu/mL of Em-resistant counts after 24 generations, they were under the detection limit (<1 log cfu/mL) after 58 generations. Therefore, higher numbers of transformed cells, i.e. more Lcn972 producing cells, seems to be required in order to produce enough Lcn972 and displace plasmid-free cells in the absence of antibiotic selection.

## Conclusions

The strong selective pressure exerted by bacteriocins can be exploited to enhance the segregational stability of recombinant plasmids when combined with their cognate immunity system, as shown in this work with the bacteriocin Lcn972. Once the Lcn972 recombinant plasmid is inside the cells, it is maintained without any obvious deleterious consequences for the cells and antibiotic pressure is no longer needed, providing a useful tool for the development of safer and more sustainable biotechnological applications of *L. lactis*.

## Methods

### Bacterial strains and growth conditions

*Lactococcus lactis* NZ9000 [34] was used as host for cloning and *L. lactis* MG1614 [35] was used as the Lcn972 susceptible strain. *Lactococci* were grown statically in M17 broth (Oxoid, Basingstoke, United Kingdom) supplemented with 0.5% (v/v) glucose (GM17) at 30°C. When needed, erythromycin was added at 5 μg/mL (GM17Em). Growth curves were started by inoculating pre-warmed GM17 with overnight cultures at an optical density at 600 nm (OD_600_) of 0.05. Growth rates (μ) were calculated from at least two independent cultures by linear regression of ln (OD_600_) versus time in the exponential phase and the generation time (g) as ln (2)/μ.

### Lcn972 susceptibility tests

Screening of transformants for bacteriocin production was done by a direct antagonism test. Isolated colonies were stabbed on GM17 (1.2% agar) plates previously inoculated with 10^5^cfu/mL of *L. lactis* MG1614 and the presence of inhibition halos was recorded after overnight incubation at 30°C. Bacteriocin activity in supernatants was quantified by the agar diffusion test using 20 μL of twofold dilutions placed in wells made on *L. lactis* MG1614 indicator plates. Arbitrary units (AU) were defined as the inverse of the last dilution that gave a clear halo and expressed by mL. The minimal inhibitory concentration (MIC) of Lcn972 for recombinant *L. lactis* was determined by the broth microdilution method as described before [17].

### Plasmid constructions

Plasmid pBL1 [19] was used as the source of the Lcn972 gene cluster and plasmids pIL252 [20] and pTLR1 [30] were used as cloning vectors. An overview of the cloned inserts is shown in Figure 1. Restriction enzymes were purchased from Takara (Otsu, Japan) and used as recommended. Plasmids from lactococcal cultures were purified with High Pure Plasmid Isolation kit (Roche, Mannheim, Germany) and DNA from agarose gels was recovered using the Illustra GFX PCR DNA and Gel Band Purification kit (GE Healthcare, Buckinghamshire, United Kingdom). Transformation of *L. lactis* NZ9000 was carried out as described by Holo and Nes [36]. To construct pLDH, the lactate dehydrogenase promoter (P_ldh_) was excised as a 289-bp *Bam*HI fragment from pRV85 [22] and used to replace the 324 bp *Bgl*II-*Bam*HI nisin promoter in pBL54 where the promoterless Lcn972 gene cluster had been previously cloned (IPLA-CSIC, laboratory collection). From the resulting plasmid, the whole Lcn972 gene cluster fused to P_ldh_ (3.6 kbp) was excised by digestion with *Afe*I and *Sma*I and ligated to pIL252 previously digested with *Sma*I, creating plasmid pLDH. The plasmid pHYB was constructed by replacing the 300 bp *Eco*RI carrying the promoter P_ldh_ of pLDH by the 194 bp *Eco*RI fragment from pBL57 containing the hybrid promoter P_hyb_[23]. pOrf4 was constructed by digesting pLDH with *Eco*RI and *Dra*III (internal to *lcn972*) to remove P_ldh_ and the 5’end of *lcn972* and subcloning of a *Eco*RI and *Dra*III digested amplicon (1.5 kbp) generated with primers orf4F and orf4R (Table 2) and pBL1 as template. PCR amplification was performed at 55°C melting temperature with the proofreading Phusion high fidelity polymerase (Fisher Scientific, Spain) as indicated by the manufacturer. *orf4* in pOrf4 was partially deleted to yield pDorf4. To this end, pOrf4 was digested by *Eco*RI-*Eco*RV to delete a 705 bp fragment covering the 5’ end of the gene. Cohesive ends were filled with the DNA polymerase I Klenow fragment (Takara, Otsu, Japan) and blunt-end ligated. Plasmid pTLR1 based in the pAK80 vector and carrying the Px-*mrfp* fusion, which encodes constitutive expression of the fluorescent mCherry protein [30] was used to construct the expression shuttle plasmid pTRL1_Lcn972 (Figure 1). To this end, a PCR reaction using pDorf4 as template and the primers SPCf and SPCr flanking the Lcn972 gene cluster (Table 2) was performed. The 3.5 kbp PCR product was digested by *Eco*RV and *Sma*I and cloned upstream of *mrfp* into the Klenow-blunted *Xho*I-digested pTRL1 plasmid. All the recombinant plasmids were checked by DNA sequencing (Macrogen, Korea).

### Segregational plasmid stability

*L. lactis* NZ9000 carrying each of the recombinant plasmids were sequentially inoculated in GM17 broth for 200 generations. Samples were taken and ten-fold dilutions were plated on GM17 agar plates to isolate individual colonies. Up to 168 colonies were checked at time intervals for resistance to erythromycin by replica-plating onto GM17Em plates and for Lcn972 production onto GM17 plates (1.2% agar) inoculated with *L. lactis* MG1614. Plates were incubated overnight at 30°C to score for growth in the presence of the antibiotic and for the development of inhibition halos, respectively. Stability of *L. lactis* NZ9000/pTRL1_Lcn972 was followed by screening for pink/white colonies after plating on GM17 agar to discriminate between mCherry expressing and non-expressing cells.

### Mapping of pOrf4 integrants

To determine the plasmid region involved in the integration of pOrf4 into the chromosome, primers orf4-F/lcn2, ery5/orf4-R, eryrepF-F/repE-R, and repE-F/repE2-R listed in Table 2 were designed and combined in PCR reactions performed with PuRe *Taq* Ready-to-Go PCR beads (GE Healthcare, Buckinghamshire, United Kingdom), using as template the plasmid pOrf4, total DNA of *L. lactis* 200 g, and total DNA of *L. lactis* 400 g. Direct sequencing of the chromosome was carried out by Secugen (Madrid, Spain) using 2.5 - 3 μg of total DNA of *L. lactis* 200 g and of *L. lactis* 400 g and the primers 201 g-F/201 g-R and 402 g-F/402 g-R, respectively.

## Competing interests

The authors declare that they do not have competing interests.

## Authors’ contributions

ABC and CR carried out the experiments. MLM and PL contributed to the pTRL1 experiments. BM conceived the work and together with AR designed, supervised, and wrote the manuscript. All authors read and approved the final manuscript.
